# Where Muscle Matters: How Regional Differences, Pain, and Gender Define Gamer Health

**DOI:** 10.3390/ijerph22050687

**Published:** 2025-04-26

**Authors:** Joanne DiFrancisco-Donoghue, Min-Kyung Jung, Matteo J. Balentine, Hallie Zwibel

**Affiliations:** 1 Department of Osteopathic Medicine, NYIT Center for Esports Medicine, College of Osteopathic Medicine, New York Institute of Technology, Old Westbury, NY 11568, USA; 2 Department of Research, College of Osteopathic Medicine, New York Institute of Technology, Old Westbury, NY 11568, USA; 3 Department of Economics, College of Arts and Sciences, Georgetown University, Washington, DC 20057, USA; 4 Department of Family Medicine, NYIT Center for Esports Medicine, College of Osteopathic Medicine, New York Institute of Technology, Old Westbury, NY 11568, USA

**Keywords:** musculoskeletal pain, lean body mass, video games, dual-energy X-ray absorptiometry, sex characteristics, ergonomics, esports, repetitive strain injury

## Abstract

Competitive gaming presents unique musculoskeletal challenges due to prolonged sitting and repetitive hand and arm movements. This study explores gender-specific regional lean body mass (LBM) differences and their associations with musculoskeletal discomfort in competitive gamers. Sixty participants (30 gamers and 30 matched controls; 15 males and 15 females in each group) underwent DXA scans to assess total and regional LBM, handgrip strength tests, and self-reported musculoskeletal pain surveys. Controls were matched for age and BMI and reported comparable academic and screen time but were not engaged in competitive gaming. Male gamers exhibited significantly reduced forearm (*p* < 0.05) and upper body LBM (*p* < 0.001), alongside lower grip strength (*p* < 0.001), compared to controls. Female gamers demonstrated lower upper body LBM (*p* = 0.01) but showed no significant differences in forearm lean mass or grip strength. In male gamers, negative correlations were observed between forearm LBM and lower back pain (r = −0.59, *p* < 0.01), highlighting the protective role of regional LBM against discomfort. Extended gaming duration was associated with increased musculoskeletal pain in both sexes (*p* < 0.05). These findings emphasize the need for targeted ergonomic interventions and physical conditioning programs to address muscle imbalances and reduce injury risk in esports athletes. Future research should focus on longitudinal and interventional designs to optimize musculoskeletal health and performance in this growing population.

## 1. Introduction

The rise of competitive video gaming has brought about unique health challenges, primarily due to prolonged sitting, repetitive muscle use, and limited physical activity. Research has demonstrated lower lean body mass (LBM), higher body fat percentages, and reduced bone mineral content (BMC) in young competitive male gamers compared to age-matched controls [1,2]. Other recent studies have linked prolonged sitting in esports players to reduced trunk muscle strength and thickness [3] and increased prevalence of lower back pain [4], suggesting parallels with the musculoskeletal decline seen in older adult populations. Furthermore, deficits in forearm lean mass have been associated with overuse injuries, including tendinopathies and ulnar neuropathies, highlighting the importance of regional muscle composition in esports injury risk [5]. Despite these risks, few studies have comprehensively evaluated regional lean mass and muscle quality differences between competitive gamers and non-gamers, and even fewer have focused on sex-specific analyses.

Body composition encompasses the proportions of fat, muscle, bone, and water in the human body, serving as a crucial indicator of overall health and fitness [6,7]. Unlike traditional weight-based measures, body composition allows for a more comprehensive assessment of health risks. Higher muscle mass and lower body fat percentages are associated with better health outcomes, emphasizing the need to evaluate both lean and fat compartments in esports athletes [8,9].

Understanding body composition is vital for injury prevention and recovery, particularly among athletes. Higher muscle mass improves strength and resilience and supports rehabilitation following injuries [10,11]. Muscle is denser than fat, meaning individuals with the same BMI (total mass/height²) can have vastly different health profiles depending on their lean-to-fat ratio [11,12].

Various measurements of lean mass (LM) have been studied to better understand an individual’s health. However, measuring LM utilizing dual X-ray absorptiometry encompasses more than just muscle mass; it also includes the mass of skin, internal organs, tendons, and other substances. Approximately 53% of LM comprises total body muscle mass [13,14]. Consequently, many researchers advocate using appendicular lean body mass (ALM) instead of total LM as a more accurate measure of skeletal muscle mass and strength [14,15,16]. Since an individual’s height influences LM, standardizing it to height, known as lean mass index (LMI), is advisable. The appendicular lean mass index (ALMI) provides a more focused approach that is particularly relevant to this study [7,8].

Despite available reference centiles for LMI and ALMI [10,17], few studies have applied these metrics to young adult esports athletes. Additionally, ALMI in relation to grip strength serves as a proxy for muscle quality (MQ), an important but poorly standardized marker reflecting a muscle’s capacity for force generation and function [10,17]. Despite the lack of a universally accepted definition, MQ is related to several dimensions. We concentrate on muscle and force-producing capacity for this investigation.

The lean mass to fat mass (LMF) ratio is another valuable measure, representing the proportion of lean tissue relative to fat mass [9,18]. This ratio shows the proportion of lean body mass—which includes muscles, bones, and organs—to fat mass [6,16]. LM distribution, particularly in the limbs, is also associated with function, recovery, and sex-based differences in muscle function and pain perception [11,19,20].

Esport players who play video games for extended periods of time and can execute up to 6oo actions per minute are at risk for musculoskeletal ailments primarily in the upper and lower back, shoulder, wrist, and hands [14]. Repetitive strain on specific muscle groups, particularly in the forearms, increases susceptibility to overuse injuries, such as tendinopathies and peripheral neuropathies [5,21].

Prior work has largely focused on male gamers and generalized body metrics. This study is the first to comprehensively examine regional lean mass, lean mass indices, and muscle quality in competitive gamers using a sex-specific lens. Specifically, the aims of this research are as follows: examine regional LM, LMI, ALMI, MQ, and LMF ratios in male and female gamers versus controls; investigate correlations between these measures and self-reported musculoskeletal pain; and identify potential sex-specific differences in these associations.

Recognizing sex-based physiological differences in muscle and pain perception is critical for designing effective, targeted interventions in esports. This study addresses that gap and provides foundational data for future longitudinal and interventional research.

## 2. Materials and Methods

The New York Institute of Technology Institutional Review Board approved this study, and all participants provided written informed consent prior to participation. This study recruited adults aged 18–28 who self-identified as male or female, as our study focused on differences in body composition and strength. Participants were not excluded based on gender identity; however, due to the scope of the study, data were analyzed within these groups. Future research should include a broader spectrum of gender identities to enhance generalizability. Inclusion of the gaming participants was required to play first-person shooter games at a “gold” ranking or higher (self-reported) and engage in PC gaming for at least three hours daily. The inclusion criteria for the control participants matched the sex and BMI of the gaming participants. Control subjects must not have been competitive gamers or self-identified as a “gamer”.

The exclusion criteria for all participants included those who had sustained hand or upper body injuries within the previous year, pregnant individuals, and anyone with contraindications to dual X-ray absorptiometry [20].

Gamers were recruited online by leveraging platforms such as Facebook^®^, Twitter^®^, Slack^®^, Twitch^®^, and Discord^®^. This approach targets specific groups and servers engaged in gaming activities. Concurrently, control subjects were recruited through social media servers and from local universities, with additional outreach on social media platforms

### 2.1. Subjects

Sixty subjects provided written informed consent, and all subjects completed the study, including thirty competitive gamers, fifteen males (age 19.6 ± 1.7), fifteen females (22.2 ± 3.7), and fifteen age-matched and BMI-matched male and female controls (age 21.2 ± 3.0, 22.7 ± 3.5) (Table 1). A priori power analysis was conducted to determine the sample size required to detect group differences. Based on key outcomes such as Appendicular Skeletal Muscle (ASM) and bone mineral density (BMD) Z-scores, large effect sizes (Cohen’s d ≈ 0.8 to 1.0) were observed. With a power of 0.8 and α = 0.05, a minimum sample size of 15 participants per group was deemed sufficient. Power calculations were performed using G*Power 3.1.

### 2.2. Measurements

Each participant underwent a body composition scan using Lunar GE^®^ dual X-ray absorptiometry (DXA) (Chicago, IL, USA). Dual-energy X-ray absorptiometry measures LM as a sum of non-fat and non-bone tissues, correlating strongly with LM values obtained through CT and MRI (r^2^ = 0.86–0.96). Its low cost, minimal time requirement, and low radiation exposure make DXA an optimal choice for LM assessment in a clinical setting [22]. Data encompassing various health metrics such as BMI, total LM, total upper and lower arm region LM, and body fat were collected from 60 participants. These participants were evenly divided into two groups of 30, one consisting of gamers and the other of non-gamers, and further stratified by sex. Following the scan, subjects performed a handgrip strength test using a Jamar^®^ Hydraulic Hand Dynamometer (Performance Health Supply Inc., Warrenville, IL, USA). One of the co-investigators administered this test and adhered to validated methods and procedures. Handgrip dynamometry is a widely employed procedure, not only as an indicator of hand and forearm strength but also as a general indicator of overall strength. Grip strength norms are typically presented as summary statistics by gender, side, and age group and may include regression equations [23]. Participants were also asked to complete the International Physical Activity Questionnaire (IPAQ-Short) to determine physical activity, as well as complete questions regarding the frequency and intensity of strength training, cardiovascular exercise, and regional pain experienced while gaming [24]. The pain data in this study were obtained using a self-reported frequency-based questionnaire developed specifically for this project. Although not a standardized or formally validated instrument, it was pilot-tested in a subsample of esports players to ensure clarity and consistency of interpretation. Future research would benefit from the inclusion of validated pain assessment tools, such as the Brief Pain Inventory or Visual Analog Scale, to improve generalizability and comparability across studies.

Based on body composition metrics, indices were calculated using the following: FMI = body fat mass (kg)/body height (m^2^); LMI = total lean mass (kg)/body height (m^2^); ALMI = appendicular lean mass (kg)/body height (m^2^); LMF ratio = total lean mass (kg)/total body fat (kg); MQ = grip strength (kg)/total lean mass (kg); appendicular MQ = grip strength (kg)/total appendicular lean mass (kg).

### 2.3. Statistical Analysis

Descriptive statistics were used to calculate the mean and standard deviation for each health metric for each group. Statistical analyses were conducted using SPSS v28. A two-way ANOVA (Sex × Group) was performed for each primary outcome (regional LM, ALMI, LMI, MQ, LMF). Interaction and main effects were examined with post hoc Bonferroni corrections where appropriate.

Additionally, we conducted a correlation analysis using Spearman’s rho correlation coefficient to examine the relationships between body composition metrics and pain metrics. The analysis was performed using IBM SPSS^®^ Statistics for Windows^®^ (V29.0, Armonk, NY, USA: IBM Corp.) and R Foundation for Statistical Computing (Vienna, Austria) [25]. Cohen’s guidelines were used to determine effect size: small effect = 0.10, medium effect = 0.30, and large effect = 0.50 or higher [26]. Multiplicity correction was not applied as all comparisons were made to study and describe the differences between the gamers and controls in males and females in an exploratory manner. A *p*-value of less than 0.05 was used to determine statistical significance.

## 3. Results

### 3.1. Total Body Composition Absolute Values, Regional LM Differences in Dominant vs. Non-Dominant Forearms in Competitive Esport Players, LMF Ratio, MQ, and BMD

Physiological differences between gamers and control groups, stratified by gender. Males and females in both groups displayed comparable demographics regarding age, BMI, and visceral fat levels. However, significant physiological discrepancies were observed between the groups. In males, gamers exhibited a higher body fat percentage (*p* = 0.03, d = 1.00), reduced total LM (*p* = 0.02, d = 0.56), total lower body LM (*p* = 0.05, d = −0.45), and lower total upper body LM (*p* < 0.001, d = 1.03). Additionally, the lowest Z-score for BMD was significantly lower in the gamer cohort (*p* = 0.02, d = 0.85) (Table 2A).

In contrast, female gamers and controls had no significant differences in body fat percentage (*p* = 0.22). However, similar to males, female gamers showed lower total LM (*p* = 0.01 d = −0.95), total upper body LM (*p* = 0.01, d = −1.17)), total lower body LM (*p* = 0.04, d = −0.82)), and total appendicular LM (*p* = 0.01). The lowest BMD Z-score mirrored the disparity seen in male gamers, which was lower in female gamers (*p* = 0.02) (Table 2A).

Significant differences were found between dominant (*p* = 0.05, d = −0.25) and non-dominant (*p* = 0.04, d = −0.34) forearm LM in males but not in females (*p* = 0.36; *p* = 0.28).

Appendicular muscle quality:

No significant difference was observed among males (*p* = 0.90), while female gamers exhibited significantly higher appendicular muscle quality than female controls (*p* = 0.02, d = 0.82).

Total LMF ratio:

A significant difference was observed among males (*p* = 0.03, d = 0.50), with gamers showing a higher LMF ratio. The difference among females was not statistically significant (*p* = 0.20).

Total MQ:

No significant differences were observed between males (*p* = 0.10) and females (*p* = 0.80).

Significant differences were more pronounced among males, with lower lean mass indexes and a higher LMF ratio. Among females, the only significant difference was in appendicular muscle quality.

These results are presented in Table 2B.

### 3.2. LMI, ALMI, Arm Lean Mass Index, FMI

Table 3 compares lean mass index (LMI), appendicular lean mass index (ALMI), and arm lean mass index between gamers, controls, and reference ranges.

Lean mass index (LMI):

A significant difference was observed between male gamers and male controls (*p* = 0.04, d = −0.56), while the difference among females did not reach statistical significance (*p* = 0.09).

Appendicular lean mass index (ALMI):

Male gamers had significantly lower AMI than male controls (*p* = 0.02, d = −1.03). The difference among females was not statistically significant (*p* = 0.10).

Arm lean mass index:

Significant differences were found in both males (*p* < 0.001, d = −0.85) and females (*p* = 0.05, d = −1.17), with gamers showing lower values compared to controls.

Fat mass index:

No significant differences were found in male and female gamers (*p* = 0.19, *p*= 0.44).

### 3.3. Grip Strength, Sit Time, and Physical Activity by Gender in Gamers and Non-Gamers

There were significant decreases in grip strength on both the dominant and non-dominant sides in male gamers compared to controls (*p* < 0.001, d = −1.36, *p* < 0.001, d = −1.37). In contrast, there was no difference in grip strength between female gamers and female controls (*p* = 0.3, *p* = 0.4). Interestingly, despite having differences in grip strength, body fat, and LM, there was no difference in self-reported cardiovascular exercise, strength training, and sitting time between male gamers and controls. However, descriptive results of the IPAQ-S revealed that only 26.7% of male gamers reported high activity, whereas male controls reported 80% high activity levels (Table 1). In contrast, female gamers showed no difference in strength but self-reported significantly less time performing cardiovascular and strength training exercises weekly (*p* = 0.04, d = −0.79, *p* = 0.01, −0.75). Female gamers also reported significantly more time spent sitting than controls (*p* = 0.01, d = 1.19). The results are presented in Table 2B. Only 20% of female gamers reported high activity on the IPAQ-S, whereas 64.3% of controls reported high activity. Only 7% of female controls reported low activity, whereas 53.3% of female gamers reported low activity. These results are presented in Table 1.

### 3.4. Hand Grip Strength and Correlations of Forearm LM

In male gamers, dominant hand grip strength showed a strong positive correlation with lower arm LM (r = 0.65, *p* < 0.001), upper arm LM (r = 0.73, *p* < 0.001), and whole arm LM (r = 0.74, *p* < 0.001).

In female gamers, dominant handgrip strength had a strong positive correlation with lower-arm LM (r = 0.65, *p* < 0.001) and whole-arm LM (r = 0.74, *p* < 0.001). However, the correlation with the upper arm LM was negligible (r = 0.03, *p* < 0.001).

### 3.5. Correlations LM to Self-Reported Regional Musculoskeletal Discomfort

#### 3.5.1. Lower Back Pain and LM

In the male gamer cohort, we found strong negative correlations between the incidence of lower back pain and measures of total LM, including specific assessments of arm and upper body LM. The correlation coefficients ranged from −0.68 to −0.77 across different measures of LM, including total LM, LM in arms (both dominant and non-dominant), upper LM in both arms, and lower LM in the left arm. Table 4 The LMF ratio negatively correlated with lower back pain (r = −0.54, *p* = 0.04). These findings suggest that higher LM levels and lower LMF ratios are associated with a lower prevalence of lower back pain in male gamers.

Negative correlations exist among female gamers, but they are generally weaker than in the male gamer group, ranging from −0.31 to −0.47.

Overall, there was a negative correlation between lower back pain and various measures of LM and the LMF ratio, with stronger correlations observed in the male gamer group. The negative correlations indicate that lower back pain increases as LM tends to decrease. In males, as the LMF ratio increases, lower back pain and upper arm pain increase. However, this was not observed in female gamers.

#### 3.5.2. Shoulder and Upper Arm Pain

Negative correlations (−0.13 to 0.70) indicate an inverse relationship between upper arm/shoulder pain and various measures of LM, including total LM, total LM in arms, total LM in both dominant and non-dominant arms, upper LM in both left and right arms, and lower LM in both left and right arms in male gamers. A moderate negative correlation between LMF ratio and upper arm pain was seen in males only (r = −0.38, *p* = 0.16)

Female gamers: weak correlations (0.06 to −0.14) suggested a limited relationship between upper arm/shoulder pain and LM measures.

#### 3.5.3. Wrist Pain and Upper Back Pain

No relationship was noted between various regions of LM or the LMF ratio and wrist pain or upper back pain in male and female gamers.

### 3.6. Differences in Indices and Correlations

#### 3.6.1. Upper Arm and Shoulder Pain

Among male gamers, significant negative correlations were observed between LMI (r = −0.51, *p* = 0.05) and AMI (r = −0.52, *p* = 0.04) with upper arm and shoulder pain. Table 5. This suggests that higher LM may be associated with reduced pain. The correlation between BMI and shoulder pain (r = −0.45, *p* = 0.09) was negative but not significant. MQ (r = 0.25, *p* = 0.37) and ALM MQ (r = 0.44, *p* = 0.10) showed positive correlations but were not significant.

No significant correlations were found between any body mass indices and upper arm or shoulder pain in female gamers. The strongest correlation was seen for LMI (r = 0.34, *p* = 0.22).

#### 3.6.2. Lower Back Pain and Body Indices

In male gamers, BMI (r = −0.76, *p* < 0.001), LMI (r = −0.77, *p* < 0.001), and AMI (r = −0.81, *p* < 0.001) all showed significant negative correlations with lower back pain. These results suggest that higher LMI and BMI are associated with reduced lower back pain in this group, with AMI having the strongest relationship. Additionally, ALM MQ (r = 0.65, *p* = 0.01) showed a significant positive correlation with lower back pain, indicating that higher muscle quality may be linked to increased pain perception.

No significant correlations were observed in female gamers. BMI and LMI showed a negative correlation with lower back pain (r = −0.41, *p* = 0.13).

### 3.7. Frequency of Play and Low Back Pain/Upper Arm Pain

**Figure 1:**
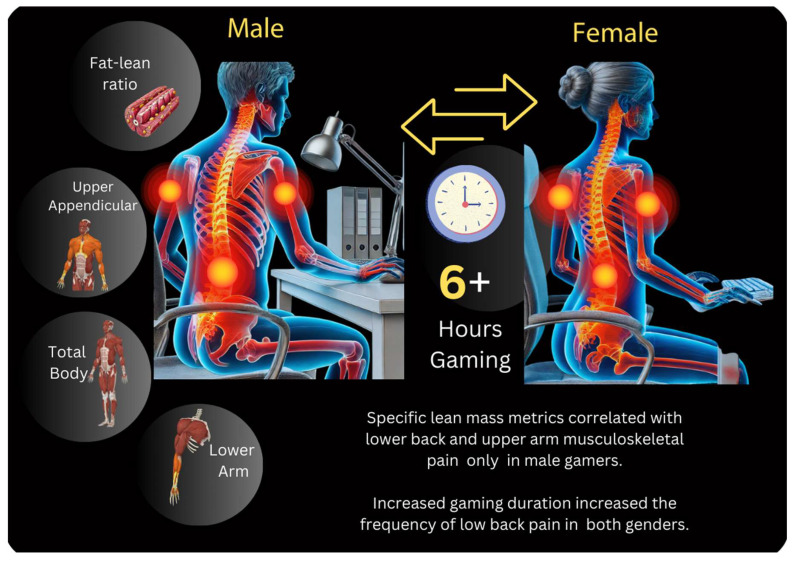
Correlations between regional lean mass, gaming hours, and musculoskeletal pain in competitive gamers.

A descriptive analysis was conducted to examine the prevalence of lower back pain among gamers based on weekly gaming duration. Percentages were used to compare the frequency of reported pain across different gaming time categories. The prevalence of lower back pain was higher among gamers who played more than six hours per week compared to those who played six hours or less. Specifically, the proportion of individuals reporting no lower back pain decreased from 60% in the ≤6 h group to 40% in the >6 h group. Conversely, the percentage of individuals experiencing back pain ‘sometimes’ increased, and reports of persistent pain (‘always’) doubled. These findings suggest a potential dose–response relationship between gaming duration and lower back pain frequency. Figure 1 displays all metrics that correlated to musculoskeletal pain by gender.

## 4. Discussion

This study is the first to use advanced body composition technology to explore the relationships between regional LM, LMF ratio, LMI, and musculoskeletal pain in gamers using a sex-specific analysis. Our findings contribute significantly to the existing literature by identifying distinct sex-specific patterns in body composition and muscle quality among the gaming population compared with age and BMI controls, underscoring the importance of recognizing physiological differences by sex in esports populations.

Male gamers exhibited significantly lower total LM, upper-arm LM bilaterally, and forearm LM than controls, along with reduced strength. In contrast, female gamers displayed no differences in body fat percentage but showed lower total LM, upper-arm LM, and bone mineral density (BMD) relative to their non-gaming counterparts. Interestingly, female gamers did not demonstrate significant differences in strength compared to controls, suggesting that the impact of gaming on muscle function may vary by sex. These observations highlight the complex interplay between gaming behavior, MQ, and functional capacity. The LMF ratios provide critical insights into MQ, revealing elevated appendicular ratios in male and female gamers. In males, higher LMF ratios in the forearms suggest reduced MQ, potentially increasing susceptibility to overuse-related musculoskeletal pain, such as symptoms associated with tendinopathies and ulnar neuropathies [27,28]. Elevated LMF ratios in female gamers, notably in the arms (0.71 vs. 0.61 in controls) and legs (0.68 vs. 0.58 in controls), may impair fine motor control, endurance, posture, and seated stability, which are all critical for prolonged gaming sessions.

While muscle quality (MQ) appears frequently throughout our analysis, it is important to clarify its definition and clinical relevance. In this study, MQ was calculated as grip strength relative to lean mass, reflecting the functional capacity of skeletal muscle tissue. Clinically, MQ is increasingly recognized as a more sensitive biomarker of musculoskeletal health than muscle mass alone, as it accounts for a muscle’s ability to generate force per unit mass [10]. Higher MQ indicates greater neuromuscular efficiency, while lower MQ may reflect sarcopenic changes, fat infiltration, or compromised muscle function despite preserved mass [10,27,28]. Although MQ lacks a universal clinical threshold, studies have demonstrated its relevance in predicting mobility, strength-related limitations, and even the risk of falls and frailty in various populations [10,27]. In the context of esports athletes, MQ is especially meaningful, as it may identify performance-limiting deficits not captured by lean mass alone.

The regional muscle mass and muscles used in mouse control provide additional context for these findings. Mouse control and clicking are highly repetitive actions that involve several key muscle groups in the forearm and hand, including the flexor carpi radialis, flexor carpi ulnaris, extensor carpi radialis longus and brevis, extensor digitorum, and pronator teres [5]. These muscles are often subjected to repetitive stress during gaming, potentially contributing to overuse-related discomfort over time [4,20,29]. This study demonstrated that male gamers had significantly lower LM in their forearms than controls, providing critical insights into the potential link between muscle composition and susceptibility to musculoskeletal pain. Reduced LM in the forearms may limit the ability of the muscles to withstand repetitive stress, predisposing gamers to conditions such as flexor and extensor tendinopathies and peripheral neuropathies, including ulnar neuropathy, due to inadequate muscular support and neural protection. A lower LM may also imply reduced muscular endurance, resulting in an earlier onset of fatigue during prolonged gaming sessions. Fatigue can lead to compensatory movements and postures, further increasing the risk of musculoskeletal discomfort.

Appendicular LMF ratios are more indicative of muscle functionality than whole-body measures, as they focus on skeletal muscle-specific changes [28], and the elevated ratios observed in this study emphasize the localized impact of sedentary gaming behaviors on muscle LMF ratio. Muscle quality and functional capacity are strongly linked to appendicular ratios, as demonstrated by their correlations with grip strength and muscular pain. Male gamers displayed stronger negative correlations between LM and lower back pain, suggesting that lean mass plays a protective role against musculoskeletal discomfort. This effect was less pronounced in females, possibly due to differences in body composition, activity patterns, or gaming styles, emphasizing the need for sex-specific interventions [16,30,31].

Our results are consistent with prior studies demonstrating reduced regional lean mass and increased musculoskeletal discomfort in sedentary or tech-based occupational populations [3,4]. Additionally, our finding that lean mass indices and muscle quality are associated with lower back pain in male gamers parallels observations in athletic and clinical populations, where diminished muscle strength and imbalanced body composition are linked to pain and performance decrements [27].

Interestingly, the lack of significant correlations between lean body mass and pain in female gamers may also be attributed to hormonal, behavioral, or psychosocial factors. Estrogen has known effects on tendon elasticity, pain modulation, and muscle recovery, potentially buffering the musculoskeletal impact of lower LBM in females. Additionally, female gamers may adopt different playing postures, take more frequent movement breaks, or engage in compensatory behaviors that mitigate repetitive strain. Psychosocial differences in pain perception and reporting could also contribute to the absence of associations seen in this group. Moreover, the lack of significant association between wrist pain and LBM, despite the repetitive nature of mouse use in gaming, suggests that wrist pain may arise more from ergonomic stressors, tendon fatigue, or cumulative overuse of smaller muscle groups rather than gross muscle mass. These findings point to the complexity of musculoskeletal pain etiology in gamers and underscore the need for future studies to explore ergonomic factors, motor control strategies, and sex-specific physiological differences.

Our findings have important clinical and research implications. Resistance training programs designed to increase LM in the arms and legs could counteract the imbalances observed in both sexes. Ergonomic adaptations, such as adjustable seating and hand support devices, may mitigate the strain in compromised muscle groups. Nutritional strategies that promote LM retention and reduce fat infiltration should be incorporated into intervention programs. Additionally, sex-specific approaches are warranted: female gamers may benefit from targeted upper body strengthening to improve muscle quality and reduce overuse-related musculoskeletal symptoms, whereas male gamers should prioritize larger core muscle groups for postural training and leg strengthening to address elevated LMF ratios.

While appendicular ratios are most relevant for evaluating muscle strength and functional outcomes, whole-body ratios remain valuable for assessing systemic health risks such as metabolic syndrome. However, focusing on regional metrics offers a more precise understanding of how gaming-specific behaviors influence muscle quality and functional capacity. This distinction is particularly important for designing effective interventions tailored to the unique physical demands of esports athletes.

Regarding body composition indices, the analysis of body composition indices and their correlation with pain in gamers revealed that fat mass index (FMI) exhibited the weakest association with pain, suggesting that fat mass does not play a significant role in postural support or musculoskeletal strain during prolonged gaming. In contrast, the Appendicular Muscle Index (AMI) demonstrated the strongest negative correlation, particularly with lower back pain in male gamers (r = −0.81, *p* < 0.001), indicating that greater limb muscle mass may enhance postural endurance and reduce musculoskeletal discomfort. Lean mass index (LMI) followed AMI in the strength of correlation, further supporting the hypothesis that greater total lean mass contributes to musculoskeletal resilience during prolonged sedentary activity. Body Mass Index (BMI), while moderately correlated with pain, lacked specificity, as it encompasses both fat and muscle mass, limiting its utility in predicting postural strain-related discomfort.

Previous research on grip strength and its role in motor control has demonstrated that improved grip strength is associated with enhanced motor control in male esports athletes, likely due to increased neuromuscular coordination and dexterity [32]. An 8-week physical training program showed improved grip strength in male gamers, supporting its role in fine motor skill development [33]. Our findings further indicate that non-dominant grip strength in males had a moderate correlation with upper arm and shoulder pain. In contrast, dominant grip strength exhibited a strong correlation with upper arm and shoulder pain and a moderate correlation with lower back pain. However, we found a low to no correlation between grip strength and these pain measures in female gamers, highlighting a potential sex-based difference in the relationship between grip strength and musculoskeletal discomfort. Notably, no intervention studies to date have specifically targeted grip strength improvements in female esports athletes, representing a gap in the literature. Future research should explore whether tailored training programs could yield similar benefits for female gamers in motor control and musculoskeletal health.

The limitations of this study include its cross-sectional design, which prevents causal inferences, and reliance on self-reported pain and activity levels, which may introduce bias. Future research should include longitudinal studies to evaluate the progression of muscle quality changes over time and to assess the long-term effects of gaming on musculoskeletal health. Interventional studies focusing on resistance training, ergonomic modifications, and nutrition are also required to determine practical strategies for mitigating the identified risks. Expanding the study population to include casual gamers and diverse demographics would enhance the generalizability of these findings. Finally, it is important to acknowledge that a higher proportion of male gamers in our sample reported elite-tier competitive status compared to females. This disparity in level of play may have influenced group comparisons and should be considered in future research designs.

Our findings underscore the importance of skeletal muscle mass, particularly in the limbs, in mitigating gaming-related musculoskeletal discomfort while suggesting that fat mass has minimal protective or aggravating effects on pain in this population. To translate these findings into practice, we recommend the development of integrated intervention models for esports athletes that include strength assessments, personalized resistance training programs, and ergonomic gaming setup evaluations. Coaches and performance staff should implement baseline body composition and muscle quality screenings, while health professionals can guide injury prevention strategies based on regional LM and MQ profiles.

## 5. Conclusions

In conclusion, this study provides novel insights into the body composition and musculoskeletal health of competitive gamers, with a particular focus on sex differences. Elevated appendicular LMF ratios and reduced regional LM highlight the localized effects of gaming-related behaviors on muscle quality and function. The findings suggest that higher lean mass—reflected in LMI, ALMI, and arm lean mass index—is associated with reduced pain in male gamers, particularly lower back pain. In contrast, no significant relationships were found in female gamers, suggesting that pain risk factors may differ by sex. Further research is needed to explore the biomechanical and lifestyle factors contributing to these differences. Tailored interventions that address these disparities are critical for promoting long-term health, performance, and resilience in esports athletes.

## Figures and Tables

**Table 1:** Demographics.

	Men Gamers	Men Controls	Women Gamers	Women Controls
	n = 15	n = 15	n = 15	n = 15
Age (SD)	19.6 ± 1.7	21.2 ± 3.0	22.3 ± 3.4	22.7 ± 3.5
Weight, kg (SD)	73.2 ± 17.4	80.3 ± 22.4	59.4 ± 12.6	61.9 ± 10.5
Height, cm (SD)	173.7 ± 9.8	180.8 ± 9.2	162.3 ± 9.1	165.1 ± 5.8
BMI (SD)	24.4 ± 5.4	24.2 ± 4.2	22.3 ± 5.7	23 ± 3.8
FMI (SD)	22.1 ± 10.3	19.8 ± 14.5	22.7 ± 10.1	20.3 ± 8.9
Right-handed (%)	86.7	73.3	93.3	100.0
Ethnicity				
African American	0.0	6.7	20.0	0.0
Asian	46.7	13.3	33.3	20.0
Hispanic	40.0	13.3	20.0	6.7
Caucasian	13.3	66.7	26.7	73.3
Physical Activity Level (IPAQ-S) (%)				
Low	33.3	0.0	53.3	7.0
Moderate	40.0	20.0	26.7	28.6
High	26.7	80.0	20.0	64.3
Primary Game (%)				
Rocket League	13.3	n/a	6.7	n/a
Valorant	26.7	n/a	0.0	n/a
Overwatch	20.0	n/a	13.3	n/a
League of Legends	6.7	n/a	20.0	n/a
Other	33.3	n/a	60.0	n/a
Casual Hours of Video Games Played Weekly (%)				
Less than 1 h	0.0	60.0	0.00	80.0
1–2 h	0.0	6.7	20.0	6.7
3–4 h	13.3	13.3	6.7	13.3
5–6 h	6.7	13.3	20.0	0.0
More than 6 h	80.0	6.7	53.3	0.0
Competitive Hours Played Weekly (%)				
Less than 1 h	0.0	n/a	53.0	n/a
1–2 h	46.7	n/a	20.0	n/a
3–4 h	6.7	n/a	13.0	n/a
5–6 h	6.7	n/a	6.7	n/a
More than 6 h	40.0	n/a	6.7	n/a

**Table 2:** (A) Body composition, muscle metrics, and bone health. (B) Muscle quality, strength, activity, and sedentary behavior.

(A)						
	Male Gamers	Male Control	*p*-Value	Female Gamers	Female Control	*p*-Value
Outcome	n = 15	n = 15		n = 15	n = 15	
Body Composition						
Age	19.6(1.7)	21.2(3.0)	0.1	22.2 (3.7)	22.7 (3.5)	0.75
BMI	24.4 (5.4)	24.2 (4.2)	0.91	22.3 (5.7)	23 (3.8)	0.84
Body Fat %	28.7 (7.1)	20.0 (10.0)	0.03 *	35.2 (8.9)	31.2 (8.5)	0.22
Fat-Free Mass, kg	52.4 (10.8)	62.9 (10.9)	0.01 *	38.4 (4.6)	42.9 (5.2)	0.02 *
Visceral Fat	0.46 (0.5)	0.5 (0.9)	0.62	0.35 (0.4)	0.25 (0.4)	0.53
Lean Body Mass (LM), kg	49.7 (22.6)	59.5 (10.6)	0.02 *	36.1 (4.3)	40.5 (4.9)	0.01 *
Total Lower Body LM, kg	17.7 (8.1)	20.5 (3.4)	0.05 *	12.3 (1.9)	13.9 (2.0)	0.04 *
Total Upper body LM, kg	6.1(2.8)	8.0 (1.5)	<0.00 *	3.5 (0.6)	4.2 (0.6)	0.01 *
Appendicular Skeletal Muscle (ASM, kg)	30.9 (7.4)	38.1 (6.6)	<0.00 *	19.9 (3.1)	23.1 (3.2)	0.01 *
Bone Density Z-Score	−1.6 (0.9)	−0.5 (1.2)	0.02 *	−1.4 (0.8)	−0.7 (0.9)	0.02 *
Non-Dominant Arm LBM, kg						
Total	3.0 (0.34)	3.9 (0.75)	<0.00 *	1.7 (0.3)	2.0 (0.3)	0.01 *
Upper Arm	1.8 (0.32)	2.6 (0.60)	<0.00 *	1.1 (0.2)	1.3 (0.2)	0.01 *
Lower Arm	1.2 (0.37)	1.3 (0.20)	0.04 *	0.7 (0.1)	0.7 (0.1)	0.28
Dominant Arm LBM, kg						
**Total**	3.1 (1.4)	4.1 (0.76)	**<0.00** *	1.8 (0.3)	2.1 (0.3)	**0.01** *
**(B)**						
**Outcomes**	**Male**	**Male**	***p*** **-Value**	**Female**	**Female Control**	***p*** **-Value**
	**Gamers**	**Control**		**Gamers**		
Appendicular Muscle Quality	6.5 (1.1)	6.5 (1.3)	0.9	9.1(2.5)	7.2(2.1)	0.02 *
Total Fat-to-Lean Mass (FLM) Ratio	0.4 (0.2)	0.3 (0.2)	0.03 *	0.6 (0.2)	0.5 (0.2)	0.2
Total Muscle Quality	0.8 (0.1)	0.8 (0.2)	0.1	1.1 (0.3)	0.7 (0.2)	0.8
Grip Strength—Dominant	86.7 (18.7)	112 (18.5)	<0.00 *	63.6 (14.1)	68.7 (12.2)	0.3
Grip Strength—Non-Dominant	79.9 (14.4)	103.7 (20.0)	<0.00 *	55.7 (10.9)	58.9 (10.5)	0.42
Cardiovascular Exercise Weekly (min)	103.9 (110.6)	158.8 (139.3)	0.27	66.4 (70.6)	118 (59.8)	0.04 *
Strength Training Weekly (min)	109 (78.5)	36.3 (132.1)	0.09	48.3 (14.4)	101.7 (99)	0.01 *
Sit Time Daily (min)	511 (211.6)	407 (187.1)	0.17	496 (138.8)	352 (101.1)	0.01 *

* Significance; all outcomes reported with standard deviation (SD).

**Table 3:** Sex and age-specific percentiles of lean mass index, appendicular lean mass index, and arm lean mass index.

Lean Mass Indexes	Male Gamers n = 15	Age/Sex Percentile	Male Control n = 15	Age/Sex Percentile	*p*-Value	Female Gamers n = 15	Age/Sex Percentile	Female Control n = 15	Age/Sex Percentile	*p*-Value
Lean Mass Index, (LMI, kg/m^2^)	16.4 (2.5)	<10th	18.1 (1.9)	20–30th	0.04 *	13.8 (2.1)	10–20th	14.9 (1.4)	~40th	0.09
Appendicular Muscle Index (AMI, kg/m^2^)	10.2 (1.7)	70th	11.6 (1.4)	80–90th	0.02 *	7.7 (1.5)	70–80th	8.5 (0.9)	~90th	0.1
Arm Lean Mass Index (kg/m^2^)	2.0 (0.3)	~30th	2.4 (0.3)	~50th	<0.00 *	1.3 (0.3)	10–20th	1.5 (0.2)	~50th	0.05 *

* Significance. Reference ranges for arm LMI are based on NHANES body composition reference values. *NHANES Body Comp arm LMI References*, Hinton BJ, et al. 2017 [8]. Reference ranges for LMI and ASMI based on Imboden et al. (2017) PLOS ONE [7].

**Table 4:** Regional lean body mass correlations to upper arm, shoulder pain, and lower back pain.

	Upper Arm Shoulder Pain						Lower Back Pain					
	Male Gamers			Female Gamers			Male Gamers			Female Gamers		
Lean Mass Region	R	CI Lower	CI Upper	R	CI Lower	CI Upper	R	CI Lower	CI Upper	R	CI Lower	CI Upper
Total Body	***−0.66***	−0.90	−0.28	−0.13	−0.61	0.43	***−0.71***	−0.92	−0.34	**−0.33**	−0.73	0.24
Total Arms	***−0.60***	−0.86	−0.11	0.06	−0.48	0.57	***−0.67***	−0.89	−0.24	**−0.38**	−0.76	0.19
Dominant Arm	*−0.58*	−0.85	−0.07	0.13	−0.43	0.61	***−0.64***	−0.88	−0.22	**−0.45**	−0.79	0.10
Non-Dominant Arm	***−0.56***	−0.84	−0.05	−0.02	−0.54	0.51	***−0.67***	−0.87	−0.17	**−0.31**	−0.72	0.26
Upper Dominant Arm	***−0.45***	−0.79	0.09	0.16	−0.40	0.63	***−0.45***	−0.81	0.05	**−0.47**	−0.80	0.07
Upper Non-Dominant Arm	***−0.51***	−0.82	0.02	−0.14	−0.62	0.41	**−0.57**	−0.81	0.03	**−0.33**	−0.73	0.24
Lower Dominant Arm	***−0.54***	−0.85	−0.08	−0.11	−0.60	0.44	**−0.77**	−0.92	−0.42	0.01	−0.52	0.53
Lower Non-Dominant Arm	***−0.52***	−0.83	−0.02	−0.11	−0.60	0.44	**−0.77**	−0.92	−0.42	0.01	−0.52	0.53

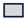
 **Bold** = Moderate negative correlation. 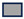 **Bold Italic** = strong negative correlation.

**Table 5:** BMI, LMI, AMI, and FMI correlations to upper arm, shoulder pain, and lower back pain.

	Upper Arm Shoulder Pain						Lower Back Pain					
	Male Gamers			Female Gamers			Male Gamers			Female Gamers		
Lean Mass Indexes	R	CI Lower	CI Upper	R	CI Lower	CI Upper	R	CI Lower	CI Upper	R	CI Lower	CI Upper
Body Mass Index (BMI)	**−0.45**	−0.78	0.08	0.16	−0.38	0.62	***−0.76***	−0.92	−0.41	**−0.41**	−0.76	0.13
Lean Mass Index (LMI, kg/m^2^)	**−0.51**	−0.81	0.01	0.34	−0.21	0.72	***−0.77***	−0.92	−0.42	**−0.41**	−0.76	0.13
MQ	0.25	−0.36	0.75	0.06	−0.47	0.56	***0.49***	−0.08	0.87	0.08	−0.45	0.57
Appendicular Muscle Index (AMI, kg/m^2^)	**−0.52**	−0.82	−0.02	0.36	−0.19	0.74	***−0.81***	−0.93	−0.51	**−0.44**	−0.78	0.10
ALM MQ	**0.44**	−0.22	0.85	−0.06	−0.56	0.47	***0.65***	0.22	0.86	−0.02	−0.50	0.50
Fat Mass Index (FMI, kg/m^2^)	**−0.44**	−0.78	0.09	0.06	−0.47	0.55	**−0.56**	−0.83	−0.07	−0.38	−0.75	0.16

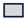
 **Bold** = moderate negative correlation. 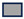 **Bold Italic** = strong negative correlation.

## Data Availability

The raw data supporting the conclusions of this article will be made available by the authors upon request.

## Abbreviations

The following abbreviations are used in this manuscript:

LM	Lean mass
BMC	Bone mineral content
BMD	Bone mineral density
LMF	Lean mass to fat mass
DXA	Dual X-ray absorptiometry
ASM	Appendicular Skeletal Muscle
BMI	Body Mass Index
ALMI	Appendicular lean mass index
LMI	Lean mass index
FMI	Fat mass index
